# Autonomic nervous system response to remote ischemic conditioning: heart rate variability assessment

**DOI:** 10.1186/s12872-019-1181-5

**Published:** 2019-09-09

**Authors:** Daniel Noronha Osório, Ricardo Viana-Soares, João Pedro Marto, Marcelo D. Mendonça, Hugo P. Silva, Cláudia Quaresma, Miguel Viana-Baptista, Hugo Gamboa, Helena L. A. Vieira

**Affiliations:** 1 LIBPhys-UNL - Laboratorio de Instrumentação, Engenharia Biomédica e Física da Radiação (LIBPhys-UNL), Departamento de Física, Faculdade de Ciências e Tecnologia da Universidade Nova de Lisboa, Monte da Caparica, 2892-516 Caparica, Portugal; 2 PLUX - Wireless Biosignals, S.A, Lisboa, Portugal; 3 CEDOC - NOVA Medical School, Faculdade de Ciências Médicas, Universidade Nova de Lisboa, Campo Mártires da Pátria, 130, 1169-056 Lisboa, Portugal; 4 Department of Neurology, Hospital Egas Moniz, Centro Hospitalar Lisboa Ocidental, Lisboa, Portugal; 5 Champalimaud Research, Champalimaud Centre for the Unknown, Lisboa, 7IT - Instituto de Telecomunicações, Lisboa, Portugal; 6 EST/IPS - Escola Superior de Tecnologia do Instituto Politécnico de Setúbal, Setúbal, Portugal; 7 iBET - Instituto de Biologia Experimental e Tecnológica, Oeiras, Portugal

**Keywords:** Remote ischemic conditioning, Electrocardiography, Heart rate variability

## Abstract

**Background:**

Remote ischemic conditioning (RIC) is a procedure applied in a limb for triggering endogenous protective pathways in distant organs, namely brain or heart. The underlying mechanisms of RIC are still not fully understood, and it is hypothesized they are mediated either by humoral factors, immune cells and/or the autonomic nervous system. Herein, heart rate variability (HRV) was used to evaluate the electrophysiological processes occurring in the heart during RIC and, in turn to assess the role of autonomic nervous system.

**Methods:**

Healthy subjects were submitted to RIC protocol and electrocardiography (ECG) was used to evaluate HRV, by assessing the variability of time intervals between two consecutive heart beats. This is a pilot study based on the analysis of 18 ECG from healthy subjects submitted to RIC. HRV was characterized in three domains (time, frequency and non-linear features) that can be correlated with the autonomic nervous system function.

**Results:**

RIC procedure increased significantly the non-linear parameter SD2, which is associated with long term HRV. This effect was observed in all subjects and in the senior (> 60 years-old) subset analysis. SD2 increase suggests an activation of both parasympathetic and sympathetic nervous system, namely via fast vagal response (parasympathetic) and the slow sympathetic response to the baroreceptors stimulation.

**Conclusions:**

RIC procedure modulates both parasympathetic and sympathetic autonomic nervous system. Furthermore, this modulation is more pronounced in the senior subset of subjects. Therefore, the autonomic nervous system regulation could be one of the mechanisms for RIC therapeutic effectiveness.

**Electronic supplementary material:**

The online version of this article (10.1186/s12872-019-1181-5) contains supplementary material, which is available to authorized users.

## Background

Organisms have developed endogenous mechanisms of defence against external aggressions. Therapeutic strategies enhancing these mechanisms can be more efficient and safer than pharmacological exogenous treatments. Hormesis or conditioning, which is a procedure by which noxious stimuli below the threshold of damage are applied to a tissue or system, promotes cellular tolerance against more severe stimuli [1]. Interestingly, it was found that conditioning could be made in a distant (remote) non-vital organ, such as a limb, but still exerting its effects in vital organs [2]. Remote ischemic conditioning (RIC) is a good example of this: in humans, it is easily applied by repetitive inflation (occlusion) and deflation (non-occlusion) of a blood pressure cuff on a limb, causing transient limb ischemia, remotely triggering self-protective pathways in the brain, heart, kidney or liver [2]. The exact mechanisms of RIC are yet not known, nevertheless, signal transmission from the remote location to target organs is hypothesized to be mediated either by humoral factors, immune cells and/or the autonomic nervous system [2].

The involvement of the autonomic nervous system was discovered when pharmacological blockade of ganglionic neurons inhibited RIC in animal models of cerebral and heart ischemia [3–5]. In experimental models, bilateral vagotomy, blockade of opioid receptors or spinal cord resection all abolished RIC effects [6–9]. Thus, evidence suggests that autonomic nervous system is involved in RIC-induced protection in experimental models.

RIC clinical studies in myocardial infarction patients have reported reductions in infarcted area, as well as an improvement of left-ventricle ejection fractions, reduction of creatinine-kinase myocardial plasma release or even ST-segment elevation resolution [10–14]. In acute ischemic stroke, two proof-of-concept clinical trials showed that RIC can increase tissue survival after 1 month [15, 16] and improve neurological outcome [17]. In patients with symptomatic intracranial arterial stenosis daily and bilateral application of RIC reduced stroke recurrence [18]. Despite some clinical evidence of RIC beneficial role, the underlying mechanisms are still unclear in humans, namely whether it acts via circulating signalling molecules or via autonomic nervous system.

Herein, a pilot study was performed to evaluate potential alterations in autonomic nervous system due to RIC. In healthy subjects, heart rate variability (HRV) was assessed through electrocardiography (ECG) during RIC procedure. HRV studies the variation between the interval of consecutive beats and it can be described by a set of features correlating these variations with the autonomic nervous system [19]. Measuring HRV before, after and during RIC procedure can be used to clarify the involvement of autonomic nervous system in the mechanisms of remote ischemic conditioning. Moreover, two subsets (young and senior) were studied because aging might impact the autonomic nervous system activity.

## Methods

### Participants

A total of 20 subjects were selected according to two age subgroups: senior and young. Senior subjects were recruited in our hospital volunteers association: “Liga dos Amigos do Hospital Sao Francisco Xavier”, while the younger subjects were recruited among Nova Medical School. The following exclusion criteria were applied: Any previous neurological disease or neurosurgical procedure, severe heart failure (NYHA class III or higher), peripheral artery disease, skin ulcers or other severe dermatological disease. Subjects were also excluded per investigator judgment if they had any unstable/severe disease. Subjects were screened for vascular risk factors (arterial hypertension, diabetes, dyslipidemia, smoking, obesity, coronary artery disease, atrial fibrillation) and current medication, which is summarized in Additional file 3: Table S1.

### Instruments

For this study, ECG and blood volume pulse (BVP) signals were recorded during the RIC procedure. BVP signal was used to confirm blood occlusion. The ECG signal was recorded using a 1-lead local differential bipolar sensor from PLUX® (Portugal), placed on the left chest, above the heart. This sensor has an input range of + − 1.5 mV, a signal band width of 0.5–100 Hz, an input impedance of >100GOhm and a common mode rejection ratio of 100 dB (Datasheet available at https://www.biosignalsplux.com/datasheets/ECG_Sensor_Datasheet.pdf). The interface with the body is made through Ag/AgCl electrodes with a solid adhesive gel. PLUX® (Portugal) also provided two BVP sensors and the data acquisition module (Datasheet available at https://biosignalsplux.com/datasheets/biosignalsplux_hub_Spec_Sheet.pdf). The data was streamed via Bluetooth to a nearby computer at 1000 Hz sampling rate and 16-bit resolution.

### Experimental design

**Fig. 1:**
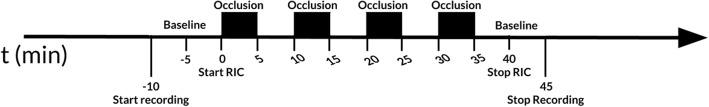
Timeline of the RIC procedure (time in minutes). Four periods of 5-min occlusion were applied with an inflated limb-cuff, each period followed by 5-min of rest (cuff deflated). Baseline recordings were taken immediately before and after RIC

The RIC protocol consisted of four cycles of 5-min ischemia/5-min reperfusion, applied to the upper limb (Fig. 1). Ischemia was performed with a blood pressure cuff inflated to above 220 mmHg or at least 20 mmHg above the subject’s systolic arterial pressure. RIC-associated adverse reactions were screened during the entire procedure.

The protocol can be summarized as follows:

1. Explanation of the study, as well as its objectives, and obtainment of participants’ informed consent;
2. Connect the ECG lead, BVP finger sensors and place the blood pressure cuff in the left arm;
3. For 10 min, record resting ECG and BVP signal from the test subject;
4. Rapidly inflate the cuff and keep the pressure high enough (about 220 mmHg) to allow the occlusion of the brachial artery. Keep the cuff inflated for 5-min;
5. Deflate the cuff. Keep the cuff deflated for another 5-min;
6. Repeat the inflation (occlusion) and deflation (non-occlusion) 3 times more;
7. Keep the cuff deflated for another 5-min;

All subjects were under the same conditions, namely: RIC procedure was applied between 9 to 10 am, in a quiet and isolated room, free from distraction. Subjects were not fasted and have taken their usual medication.

### Data analysis

#### QRS detection

According to [19], the calculation of HRV features should be done in either 24 h or 5-min intervals. Since each RIC step has a 5-min interval, each test subject’s ECG was manually segmented in 5-min segments, using the signal from the BVP as reference. After segmentation, the Pan-Tompkins algorithm [20] was applied for R-peak detection.

#### Heart rate variability features

The features assessed from the HRV can be divided in three domains: time, frequency and non-linear domain.

In the time domain, the most commonly used features are the mean R-R interval, median, root mean square of successive differences (rMSSD) and the pNN50 [19]. Using the position of the R-peaks, the difference between two consecutive beats was calculated to obtain the interval between beats, their mean, median and standard deviation. Then pNN50 and rMSSD were calculated. The pNN50 is the ratio between the number of times that changes in successive R-R interval falls outside a 50 ms threshold (NN50) by the total number of R-R interval [21, 22]. Both rMSSD and pNN50 reflect parasympathetic (vagal) activity. In fact, a decrease of pNN50 percentage suggests a lower parasympathetic activity; and rMSSD correlates with short-term HRV activity, which is used to estimate vagal-mediated changes reflected in HRV.

In the frequency domain, the normalized low-frequency (LF) and high-frequency (HF) power spectra were calculated, as well as their ratio. The literature specifies that the LF spectrum covers frequencies from 0.04 Hz to 0.15 Hz, while the HF spectrum ranges from 0.15 Hz to 0.4 Hz [19]. Since R-R interval is not a perfectly sampled event, the tachogram must be resampled to be an equally sampled signal. This can be achieved by interpolating the signal to a higher frequency [19, 23]. For this study, the inter-beat interval signal was re-sampled at 10 Hz using a cubic spline interpolation and the mean value was subtracted from the signal (the DC component of the signal). Isolated ectopic beats were corrected by linear interpolation. To get the frequency power spectral density (PSD), the Welch method [24] was used, with a 256-sample Hanning window. The normalized LF and HF power bands were obtained by dividing the power spectrum in each window by the total power minus the VLF spectral power (< 0.04 Hz) [19].

HF band reflects faster changes of heart rate, which are associated with parasympathetic activity. In fact, parasympathetic activity produces responses that have a higher frequency compared to those from sympathetic branch [25]. The LF spectrum is a combination of both the fast vagal response (part of the parasympathetic nervous system) and the slow sympathetic response to baroreceptors stimulation [26, 27]. The LF/HF ratio can also be used to predict the sympathetic/vagal balance [19]. Nevertheless, there is no consensus about the contributionof the sympathetic and parasympathetic branch in the LF band [28, 29].

Finally, in the non-linear domain, the Poincaré plot is used to describe the nature of R-R interval fluctuations, by plotting a certain R-R interval (R-Rn) versus the next one (R-Rn + 1) [30]. The resulting plot can be usually fitted into an ellipse and thus this method is used to describe the Poincaré plot. The ellipse axis associated with its width is SD1, which is a linear scaling of the standard deviation of successive differences, an important short-term measure of HRV. While, ellipse length corresponds to SD2 axis, which reflects the long-term HRV being more consistent than the standard deviation of successive R-R interval [30]. The Poincaré descriptors, SD1 and SD2, were calculated using Eqs. 1 and 2 developed by Brennan and colleagues [30], which were obtained using an ellipse fitting technique:

1 $$ SD{1}^2=\frac{1}{2} Var\left({RR}_n-{RR}_{n+1}\right)=\frac{1}{2}{SDSD}^2 $$

2 $$ SD{2}^2=2{SDRR}^2-\frac{1}{2}{SDSD}^2 $$

In Eq. 1, Var represents the variance and SDSD is the standard deviation of successive differences, calculated by getting the differences between a pair of beats and the immediately next pair.

In Eq. 2, SDRR is the standard deviation of the R-R intervals.

SD1 is mostly influenced by the parasympathetic activity, while SD2 is influenced by both the sympathetic and parasympathetic activities.

#### Features analysis

The analysed parameters were summarized into tables and filtered by age. In Additional file 1: Figure S1 there are boxplot graphs representing each 5 min-interval of the entire procedure for global population for all analysed HRV features. Finally, the first and last 10 min of each table were used to compare the effects of RIC in the entire sample and in each age group.

#### Statistical analysis

The non-parametric Wilcoxon signed-rank test was used to statistically analyse the RIC procedure effect on the different subjects. For each HRV parameter, it was analysed and compared: (i) sample pairs for occlusion and non-occlusion intervals and (ii) sample pairs for the first 10 min before the procedure and the 10 min after the last occlusion. Furthermore, same analysis was done in young and senior population subsets.

The used significance level was 0.05 (*p* < 0.05). Out of the 18 test subjects, 1 of the tested subjects had to be removed from the statistical analysis due to the lack of one time interval (before procedure). The correlation studies between changes in SD2 and in pNN50 and rMSSD were also done by non-parametric Wilcoxon test.

All calculation and data processing were done using programming language Python (v. 3.6.4) and using the following libraries: NumPy (v. 1.10.4), SciPy (v. 0.16.1) and Nova instrumentation (version 1.0).

## Results

### Subjects

Among the 20 subjects initially included, 10 in each age subgroup, two of the senior subjects were excluded from our analysis due to noisy segments, not allowing the R peak detection. On total (*n* = 18), 11 (61.1%) were female, and mean age was 47.0 ± 21.9 years. For detailed information on vascular risk factors and current medication see Additional file 3: Table S1.

### HRV features before and after RIC procedure

HRV features were analysed at 10 min before the procedure and 10 min after the last occlusion for assessing whether four cycles of RIC can modulate autonomic nervous system. All calculated values and statistical analysis (Wilcoxon signed-rank test) are represented in Additional file 4: Table S2 for the entire subject population. Additional files 5 and 6: Tables S3 and S4 represent senior and young subsets, respectively.

During aging, there is a decrease of endogenous response to stress and organism defences. Thus the use of two subsets (young and senior) can eventually disclose potential different responses of autonomic nervous system to RIC and putative novel mechanisms associated with aging processes. Furthermore, co-morbidities associated with aging such as risk of cardiovascular disease or Diabetes Mellitus might also influence autonomic nervous system response.

**Fig. 2:**
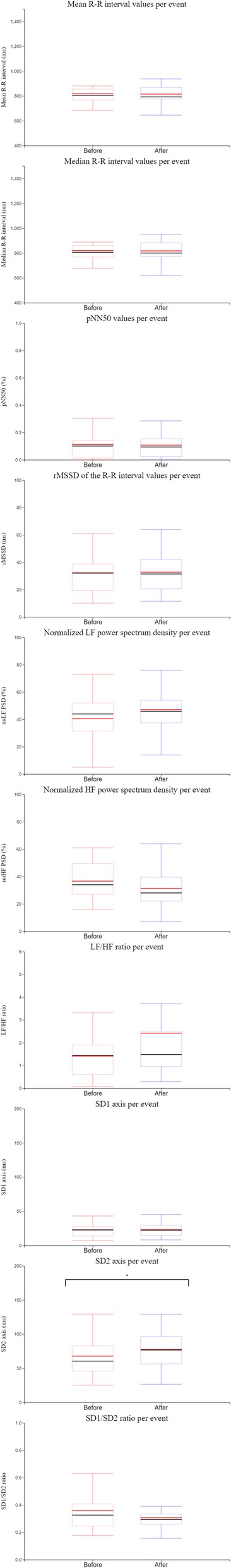
Global analysis of HRV’s features before and after RIC procedure. Each graph corresponds to one HRV feature: Mean R-R intervals (ms); median R-R intervals (ms); percentage of intervals falling outside a 50 ms difference, pNN50 (%); root mean square of successive differences of the R-R interval values per event (rMSSD (ms); normalized low frequency power spetrum density, nuLF PSD (%); normalized high frequency power spectrum density, nuHF PSD (%); LF and HF normalized power spectrum density ratio, LF/HF; SD1 axis of the Poincaré plot, SD1 axis (ms); SD2 axis of the Poincaré plot, SD2 axis (ms) and SD1/SD2 per event. Red lines correspond to mean value, black lines correspond to median value and * *p*-value < 0.05

**Fig. 3:**
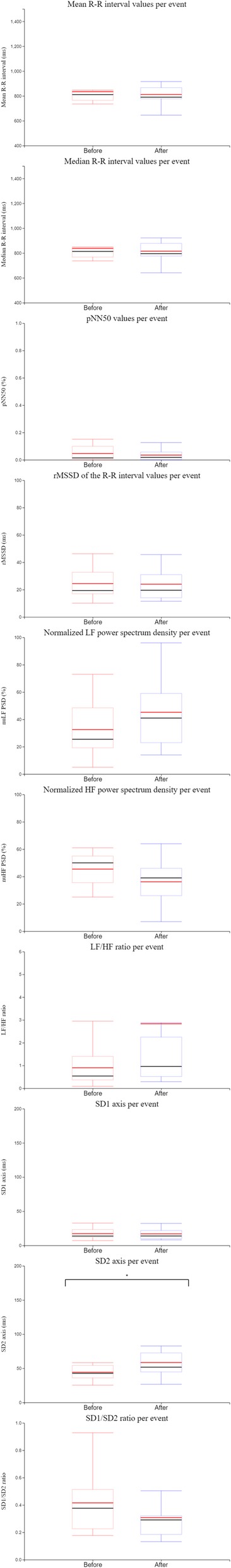
Senior subset analysis of HRV’s features before and after RIC procedure. Each graph corresponds to one HRV feature: Mean R-R intervals (ms); median R-R intervals (ms); percentage of intervals falling outside a 50 ms difference, pNN50 (%); root mean square of successive differences of the R-R interval values per event (rMSSD (ms); normalized low frequency power spetrum density, nuLF PSD (%); normalized high frequency power spectrum density, nuHF PSD (%); LF and HF normalized power spectrum density ratio, LF/HF; SD1 axis of the Poincaré plot, SD1 axis (ms); SD2 axis of the Poincaré plot, SD2 axis (ms) and SD1/SD2 per event. Red lines correspond to mean value, black lines correspond to median value and * *p*-value < 0.05

**Fig. 4:**
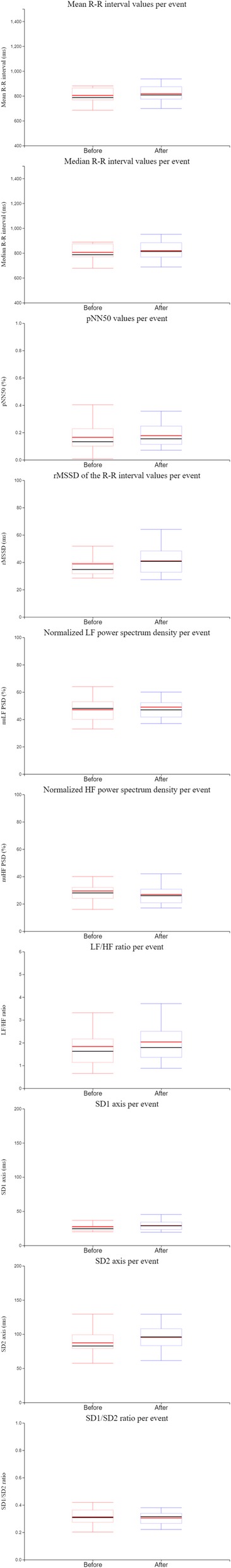
Young subset analysis of HRV’s features before and after RIC procedure. Each graph corresponds to one HRV feature: Mean R-R intervals (ms); median R-R intervals (ms); percentage of intervals falling outside a 50 ms difference, pNN50 (%); root mean square of successive differences of the R-R interval values per event (rMSSD (ms); normalized low frequency power spetrum density, nuLF PSD (%); normalized high frequency power spectrum density, nuHF PSD (%); LF and HF normalized power spectrum density ratio, LF/HF; SD1 axis of the Poincaré plot, SD1 axis (ms); SD2 axis of the Poincaré plot, SD2 axis (ms) and SD1/SD2 per event. Red lines correspond to mean value, black lines correspond to median value and * *p*-value < 0.05

The non-linear feature SD2 is the single parameter that significantly increases after RIC procedure in both global (Fig. 2 and Additional file 4: Table S2) and senior subset (Fig. 3 and Additional file 5: Table S3). While in the young subset, SD2 does not significantly change (Fig. 4 and Additional file 6: Table S4). SD2 is associated with long-term HRV, which is a combination of both the fast vagal response (parasympathetic nervous system) and the slow sympathetic response to the baroreceptors stimulation. Thus, one can propose that RIC might play a role in both sympathetic and parasympathetic activities. Concerning time and frequency domain parameters, no significant statistic differences were found before and after RIC procedure.

Because the sample size is limited and in order to further evaluate the role of RIC on autonomic nervous system, correlations between key features were also performed for all subjects. For each subject and feature the difference between its value before and after RIC procedure was calculated. Then, the differences between features were correlated. Changes in SD2 were significantly and positively correlated with changes in pNN50 and rMSSD. These results reinforce the RIC involvement of parasympathetic system via vagal response, since SD2 positively correlates with pNN50 (r = 0.45 with *p* = 0.03) and with rMSSD (r = 0.54 with *p* = 0.01), which are both associated with parasympathetic system, being rMSSD via vagal response. Nevertheless, one cannot exclude the involvement of sympathetic activity, since SD2 is related with both branches of autonomic nervous system.

### HRV features in non-occlusion vs occlusion intervals

HRV analysis during non-occlusion and occlusion intervals reveal no difference in time, frequency or non-linear domain features in senior or young subset analysis (Additional files 5 and 6: Tables S3 and S4). Nevertheless, in global analysis during non-occlusion period there is an increase on time domain parameter rMSSD (Additional file 2: Figure S2 and Additional file 4: Table S2). Thus, during non-occlusion period there might be an increase on parasympathetic activity, since rMSSD is associated with short-term HRV and with vagal response.

## Discussion

In this pilot study, the role of RIC on autonomic nervous system was assessed by analysing several HRV parameters before and after RIC procedure, namely time, frequency and non-linear domain features. Among the measured and analysed parameters, only SD2 is significantly altered due to RIC procedure. This change is positively and significantly correlated with changes in pNN50 and rMSSD, in line with the proposed biological meaning of these features. This increase in SD2 reflects an increase in heart rate variability. In fact, HRV has been associated with health-promoting conditions and can even present a prognostic value [31].

SD2 increase appears to be higher in the case of senior subset. Accordingly, age appears to influence the response of autonomic nervous system to RIC. Moreover, before RIC procedure SD2 parameter is much lower (44.309 ms) in senior subset (Additional file 5: Table S3) than SD2 parameter (86.350 ms) in young one (Additional file 1: Table S4). Thus, the more pronounced difference in SD2 parameter found in senior group could be due to a lower basal autonomic nervous activity. Furthermore, multiple studies state that the HRV parameters decrease with age [32–34]. Indeed, when parameters were analysed before RIC procedure, which indicates HRV basal levels, pNN50, rMSSD, SD2 and LF features are higher for young subset than for senior subset, accordingly with Antelmi and colleagues [32] that have shown HRV decreases with age. In addition, the risk of cardiovascular diseases increases with age, which might also decrease the response of autonomic nervous system. In particular Diabetes Mellitus (DM) is known to be associated with a decrease in HRV and it is thought to be related with the deleterious effects of sucrose in the nerves leading to an autonomic cardiac neuropathy. However, it is also known that the severity of variability reduction is related to bad glycemic control and higher HbA1c levels [35]. Although a differential confounding role of diabetes cannot be excluded, only 2 subjects (11.1%) presented a diagnosis of type 2 DM (T2DM). Moreover, these subjects had a proper glycemic control, and also had no clinical evidence of neurological dysfunction. Thus, it is not expectable this group to significantly disrupt the final findings. Finally, in the literature there are no definitive HRV parameters that can be used as an optimal biomarker to assess CVD. However, there is evidence pointing to the correlation between reduced HRV and increase risk of CVD, in particular concerning the following parameters: SDNN and nuLF and nuHF [19, 36, 37].

Furthermore, no difference in HRV was found whenever gender is considered (data not shown). In fact, in individuals older than 60 years-old, the differences in the HRV features between genders are negligible [32] due to menopause and lower oestrogen levels in women [38]. Although HRV vary immensely due to circadian rhythm [39], RIC procedure was applied always at the same hour and in a short time window (total procedure duration 60 min). Thus, daily changes might not play a key role in variations found in heart rate parameters. Finally discomfort and pain could be confounding factors able to regulate autonomic nervous system. Out of 18 subjects only 2 subjects express mild discomfort and one a mild paresthesia. Thus, the found changes in autonomic nervous system must be due to RIC procedure and not to pain or discomfort.

It was expected to find significant differences in HRV parameters during the alternation of occlusion and non-occlusion procedures, since there is disturbance introduced into the organism. Nevertheless, in global analysis only rMSSD parameter increased during non-occlusion period, suggesting higher parasympathetic activity.

RIC-induced cytoprotection can be promoted by: (i) modulation of autonomic nervous system, (ii) production and release of bio-molecules and/or (iii) immune cells signalling. In fact, in experimental models RIC also mediates distant organ protection by the release of blood-borne factors. In mice, RIC increases nitric oxide levels, which induces vasodilation and increases cerebral blow flow, besides protecting mitochondria from oxidative stress [40]. Other autacoids have been detected as possible mediators of RIC, for instance adenosine, bradykinin or calcitonin gene-related peptide [41]. In plasma from conditioned animals, SDF-1, IL-10 and microRNA-144 have been detected, miRNA-144 was also detected in human subjects but their actual role in RIC remains to be elucidated [41–44]. Finally, modulation of autonomic nervous system by RIC can be directly due to mechanical disruption of blood flow followed by reperfusion or by the release of blood borne factors. Therefore, future research on RIC will clarify the involvement of each factor (either humoral, neural or immune) in the RIC action protective mechanisms in distant organs.

## Limitations of the study

The first limitation concerns the sample size. In fact, the present work is a pilot study to assess the potential role of autonomic nervous system in RIC and to further explore the underlying mechanisms of RIC on distant organ protection. Further studies with increased number of subjects are crucial to deeper assess the role of autonomic nervous system. The second limitation is the time window of ECG recording and HRV analysis. RIC-induced modulation of autonomic nervous system was only assessed during RIC procedure and at short periods (10 min) before and after it. Therefore, further studies must be performed to assess a potential second window of autonomic nervous system modulation, namely at 2-3 h and/or 24 h following RIC procedure. Finally, more frequent RIC procedures (such as daily frequency) may also amplify the effect of RIC on sympathetic and parasympathetic activities.

## Conclusions

Electrocardiography (ECG) was used to study the effects of remote ischemic conditioning (RIC) in autonomic nervous systems of healthy subjects. RIC procedure significantly increased the non-linear parameter SD2. Finally, this data suggests that autonomic nervous system involvement could be one of the mechanisms for RIC therapeutic effectiveness.

## Additional files

Additional file 1: **Figure S1.** Global population boxplots for each phase of the RIC procedure. Each graph corresponds to one HRV feature: Mean R-R intervals (ms); median R-R intervals (ms); percentage of intervals falling outside a 50 ms difference, pNN50 (%); root mean square of successive differences of the R-R interval values per event (rMSSD (ms); normalized low frequency power spetrum density, nuLF PSD (%); normalized high frequency power spectrum density, nuHF PSD (%); LF and HF normalized power spectrum density ratio, LF/HF; SD1 axis of the Poincaré plot, SD1 axis (ms); SD2 axis of the Poincaré plot, SD2 axis (ms) and SD1/SD2 per event. (TIFF 293 kb)

Additional file 2: **Figure S2.** Global population boxplots comparing occlusion and non-occlusion intervals. Each graph corresponds to one HRV feature: Mean R-R intervals (ms); median R-R intervals (ms); percentage of intervals falling outside a 50 ms difference, pNN50 (%); root mean square of successive differences of the R-R interval values per event (rMSSD (ms); normalized low frequency power spetrum density, nuLF PSD (%); normalized high frequency power spectrum density, nuHF PSD (%); LF and HF normalized power spectrum density ratio, LF/HF; SD1 axis of the Poincaré plot, SD1 axis (ms); SD2 axis of the Poincaré plot, SD2 axis (ms) and SD1/SD2 per event. Red lines correspond to mean value, black lines correspond to median value and * *p*-value < 0.05. (TIFF 129 kb)

Additional file 3: **Table S1.** Subjects baseline characteristics: Demographics, relevant cardiovascular risk factors and medication. (PDF 44 kb)

Additional file 4: **Table S2.** Global population analysis for the first and last 10 min and occlusion and non-occlusion intervals. For the first and last 10 min analysis, the mean values are presented as well as a comparison between them and the *p*-value for the Wilcoxon signed-rank test. For the occlusion and non-occlusion interval analysis, the mean values are presented as well as a comparison between them and the *p*-value for the Wilcoxon signed-rank test. (PDF 60 kb)

Additional file 5: **Table S3.** Senior population analysis for the first and last 10 min and occlusion and non-occlusion intervals. For the first and last 10 min analysis, the mean values are presented as well as a comparison between them and the *p*-value for the Wilcoxon signed-rank test. For the occlusion and non-occlusion interval analysis, the mean values are presented as well as a comparison between them and the *p*-value for the Wilcoxon signed-rank test. (PDF 60 kb)

Additional file 6: **Table S4.** Young population analysis for the first and last 10 min and occlusion and non-occlusion intervals. For the first and last 10 min analysis, the mean values are presented as well as a comparison between them and the *p*-value for the Wilcoxon signed-rank test. For the occlusion and non-occlusion interval analysis, the mean values are presented as well as a comparison between them and the *p*-value for the Wilcoxon signed-rank test. (PDF 60 kb)

## Data Availability

The datasets used and analysed during the current study are available from the corresponding author on reasonable request.

## Abbreviations

BVP: Blood volume pulse
ECG: Electrocardiography
HF: High frequency
HRV: Heart rate variability
LF: Low frequency
PSD: Power spectrum density
RIC: Remote ischemic conditioning
R-R: Interval between two consecutive beats
VLF: Very low frequency
