# Supplementary figures and images for: Autonomic nervous system response to remote ischemic conditioning: heart rate variability assessment
Source: BMC Cardiovasc Disord. 2019 Sep 9;19:211. doi: 10.1186/s12872-019-1181-5 (PMC6734354; doi:10.1186/s12872-019-1181-5)

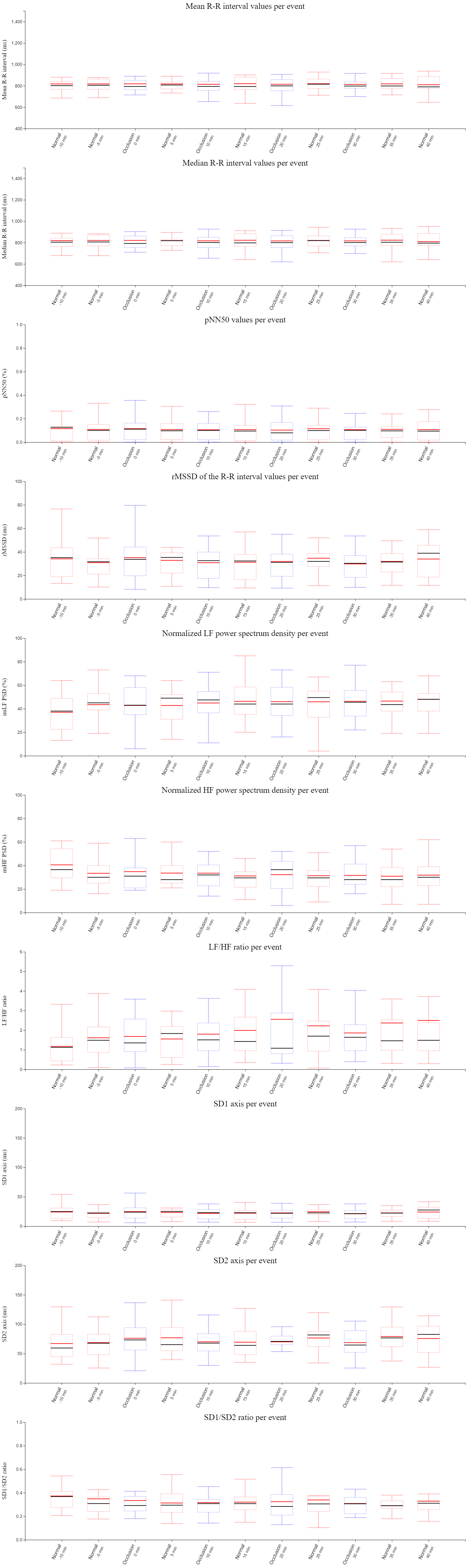

Supplement: Supplementary file 1 — Figure S1. Global population boxplots for each phase of the RIC procedure. Each graph corresponds to one HRV feature: Mean R-R intervals (ms); median R-R intervals (ms); percentage of intervals falling outside a 50 ms difference, pNN50 (%); root mean square of successive differences of the R-R interval values per event (rMSSD (ms); normalized low frequency power spetrum density, nuLF PSD (%); normalized high frequency power spectrum density, nuHF PSD (%); LF and HF normalized power spectrum density ratio, LF/HF; SD1 axis of the Poincaré plot, SD1 axis (ms); SD2 axis of the Poincaré plot, SD2 axis (ms) and SD1/SD2 per event. (TIFF 293 kb) [file 12872_2019_1181_MOESM1_ESM.tiff]

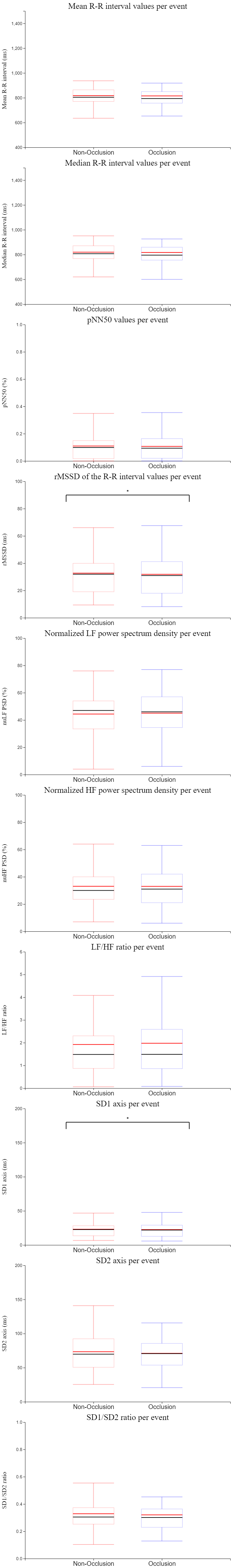

Supplement: Supplementary file 2 — Figure S2. Global population boxplots comparing occlusion and non-occlusion intervals. Each graph corresponds to one HRV feature: Mean R-R intervals (ms); median R-R intervals (ms); percentage of intervals falling outside a 50 ms difference, pNN50 (%); root mean square of successive differences of the R-R interval values per event (rMSSD (ms); normalized low frequency power spetrum density, nuLF PSD (%); normalized high frequency power spectrum density, nuHF PSD (%); LF and HF normalized power spectrum density ratio, LF/HF; SD1 axis of the Poincaré plot, SD1 axis (ms); SD2 axis of the Poincaré plot, SD2 axis (ms) and SD1/SD2 per event. Red lines correspond to mean value, black lines correspond to median value and * p-value < 0.05. (TIFF 129 kb) [file 12872_2019_1181_MOESM2_ESM.tiff]
