# Supplementary material for: Autonomic nervous system response to remote ischemic conditioning: heart rate variability assessment
Source: BMC Cardiovasc Disord. 2019 Sep 9;19:211. doi: 10.1186/s12872-019-1181-5 (PMC6734354; doi:10.1186/s12872-019-1181-5)
Supplement: Supplementary file 3 — Table S1. Subjects baseline characteristics: Demographics, relevant cardiovascular risk factors and medication. (PDF 44 kb) [file 12872_2019_1181_MOESM3_ESM.pdf]

	Included subjects (n=18)	Senior subjects (n=8)	Young subjects (n=10)
**Demographics**			
**Age, years**	47.0 ± 21.9	69.6 ± 7.6	28.9 ± 6.3
**Female sex**	11 (61.1)	6 (75.0)	5 (50)
**Cardiovascular risk factors**			
**Arterial Hypertension**	5 (27.8)	5 (62.5)	0 (0)
**Diabetes**	2 (11.1)	2 (25.0)	0 (0)
**Dyslipidemia**	6 (33.3)	6 (75.0)	0 (0)
**Smoking**	3 (16.7)	2 (25.0)	1 (10)
**Obesity**	1 (5.6)	1 (12.5)	0 (0)
**Coronary artery disease**	0 (0)	0 (0)	0 (0)
**Atrial fibrillation**	0 (0)	0 (0)	0 (0)
**Current medication**			
**Beta-blocker**	1 (5.6)	1 (12.5)	0 (0)
**Alfa-blocker**	1 (5.6)	1 (12.5)	0 (0)
**Calcium channel blockers**	1 (5.6)	1 (12.5)	0 (0)

Values are presented as mean ± standard deviation or numbers (proportions).
