# Supplementary material for: Autonomic nervous system response to remote ischemic conditioning: heart rate variability assessment
Source: BMC Cardiovasc Disord. 2019 Sep 9;19:211. doi: 10.1186/s12872-019-1181-5 (PMC6734354; doi:10.1186/s12872-019-1181-5)
Supplement: Supplementary file 4 — Table S2. Global population analysis for the first and last 10 min and occlusion and non-occlusion intervals. For the first and last 10 min analysis, the mean values are presented as well as a comparison between them and the p-value for the Wilcoxon signed-rank test. For the occlusion and non-occlusion interval analysis, the mean values are presented as well as a comparison between them and the p-value for the Wilcoxon signed-rank test. (PDF 60 kb) [file 12872_2019_1181_MOESM4_ESM.pdf]

Table S2 – Global population analysis for the first and last 10 minutes and occlusion and non-occlusion intervals

//

		Global			
		First 10 minutes Mean	Last 10 minutes Mean	First vs Last	Wilcoxon signed-rank test p-value: Before - After pairs
**Time Features**	Mean R-R Interval (ms)	819,069	834,266	Last higher	0,076
	Median R-R Interval (ms)	821,868	837,588	Last higher	0,107
	pNN50 (%)	11,351	11,670	Last higher	0,619
	rMSSD (ms)	32,430	34,152	Last higher	0,435
**Frequency Features**	nuLF PSD (%)	40,971	44,029	Last higher	0,193
	nuHF PSD (%)	36,176	32,912	First higher	0,107
	nuLF/nuHF ratio	1,424	1,644	Last higher	0,149
**Non-linear Features**	SD1 axis (ms)	22,931	24,148	Last higher	0,435
	SD2 axis (ms)	69,039	78,627	Last higher	**0,044**
	SD1/SD2	0,352	0,316	First higher	0,149

Mean R-R Interval: mean value of the time difference between beats; **Median R-R Interval**: median value of the time difference between beats; **pNN50**: percentage of beats that differ more than 50ms from the previous; **rMSSD**: root mean square of the successive differences; **nuLF PSD**: normalized power spectrum of the 0.04 to 0.15Hz band; **nuHF PSD**: normalized power spectrum of the 0.15 to 0.4 Hz band; **nuLF/nuHF**: ratio between the bands; **SD1 axis**: non-linear feature associated with short-term changes; **SD2 axis:** non-linear feature associated with long-term changes and **SD1/SD2**: ratio between axis.

Supplementary table 2 (cont.)

		Non-occlusion intervals Mean	Occlusion intervals Mean	Non-Occlusion vs Occlusion	Wilcoxon signed-rank test p-value: Non-Occlusion - Occlusion
**Time Features**	Mean R-R Interval (ms)	827,052	823,577	Non-occlusion higher	0,210
	Median R-R Interval (ms)	829,437	825,779	Non-occlusion higher	0,177
	pNN50 (%)	11,541	11,112	Non-occlusion higher	0,084
	rMSSD (ms)	33,639	32,572	Non-occlusion higher	**0,044**
**Frequency Features**	nuLF PSD (%)	42,690	43,588	Occlusion higher	0,586
	nuHF PSD (%)	33,872	33,824	Non-occlusion higher	0,868
	nuLF/nuHF ratio	1,583	1,706	Occlusion higher	0,723
**Non-linear Features**	SD1 axis (ms)	23,785	23,031	Non-occlusion higher	**0,044**
	SD2 axis (ms)	74,078	71,451	Non-occlusion higher	0,084
	SD1/SD2	0,335	0,327	Non-occlusion higher	0,332

Mean R-R Interval: mean value of the time difference between beats; **Median R-R Interval**: median value of the time difference between beats; **pNN50**: percentage of beats that differ more than 50ms from the previous; **rMSSD**: root mean square of the successive differences; **nuLF PSD**: normalized power spectrum of the 0.04 to 0.15Hz band; **nuHF PSD**: normalized power spectrum of the 0.15 to 0.4 Hz band; **nuLF/nuHF**: ratio between the bands; **SD1 axis**: non-linear feature associated with short-term changes; **SD2 axis:** non-linear feature associated with long-term changes and **SD1/SD2**: ratio between axis.
