# Supplementary material for: Autonomic nervous system response to remote ischemic conditioning: heart rate variability assessment
Source: BMC Cardiovasc Disord. 2019 Sep 9;19:211. doi: 10.1186/s12872-019-1181-5 (PMC6734354; doi:10.1186/s12872-019-1181-5)
Supplement: Supplementary file 5 — Table S3. Senior population analysis for the first and last 10 min and occlusion and non-occlusion intervals. For the first and last 10 min analysis, the mean values are presented as well as a comparison between them and the p-value for the Wilcoxon signed-rank test. For the occlusion and non-occlusion interval analysis, the mean values are presented as well as a comparison between them and the p-value for the Wilcoxon signed-rank test. (PDF 60 kb) [file 12872_2019_1181_MOESM5_ESM.pdf]

Table S3 - Senior population analysis for the first and last 10 minutes and occlusion and non-occlusion intervals

		Senior			
		First 10 minutes Mean	Last 10 minutes Mean	First vs Last	Wilcoxon signed-rank test p-value: Before - After pairs
**Time Features**	Mean R-R Interval (ms)	834,475	854,718	Last higher	0,128
	Median R-R Interval (ms)	836,929	859,607	Last higher	0,176
	pNN50 (%)	4,655	4,298	First higher	0,866
	rMSSD (ms)	24,387	26,096	Last higher	0,866
**Frequency Features**	nuLF PSD (%)	32,571	38,143	Last higher	0,310
	nuHF PSD (%)	45,429	40,714	First higher	0,310
	nuLF/nuHF ratio	0,900	1,158	Last higher	0,398
**Non-linear Features**	SD1 axis (ms)	17,244	18,452	Last higher	0,866
	SD2 axis (ms)	44,309	59,366	Last higher	**0,018**
	SD1/SD2	0,416	0,329	First higher	0,128

Mean R-R Interval: mean value of the time difference between beats; **Median R-R Interval**: median value of the time difference between beats; **pNN50**: percentage of beats that differ more than 50ms from the previous; **rMSSD**: root mean square of the successive differences; **nuLF PSD**: normalized power spectrum of the 0.04 to 0.15Hz band; **nuHF PSD**: normalized power spectrum of the 0.15 to 0.4 Hz band; **nuLF/nuHF**: ratio between the bands; **SD1 axis**: non-linear feature associated with short-term changes; **SD2 axis:** non-linear feature associated with long-term changes and **SD1/SD2**: ratio between axis.

Table S3 (cont.)

		Non-occlusion intervals Mean	Occlusion intervals Mean	Non-Occlusion vs Occlusion	Wilcoxon signed-rank test p-value: Non-Occlusion - Occlusion
**Time Features**	Mean R-R Interval (ms)	842,691	833,762	Non-occlusion higher	0,176
	Median R-R Interval (ms)	845,401	835,339	Non-occlusion higher	0,176
	pNN50 (%)	4,310	4,069	Non-occlusion higher	0,237
	rMSSD (ms)	25,637	24,216	Non-occlusion higher	0,310
**Frequency Features**	nuLF PSD (%)	35,629	36,143	Occlusion higher	0,612
	nuHF PSD (%)	42,058	41,000	Non-occlusion higher	0,612
	nuLF/nuHF ratio	1,096	1,191	Occlusion higher	0,866
**Non-linear Features**	SD1 axis (ms)	18,128	17,123	Non-occlusion higher	0,310
	SD2 axis (ms)	51,500	48,099	Non-occlusion higher	0,176
	SD1/SD2	0,371	0,353	Non-occlusion higher	0,237

Mean R-R Interval: mean value of the time difference between beats; **Median R-R Interval**: median value of the time difference between beats; **pNN50**: percentage of beats that differ more than 50ms from the previous; **rMSSD**: root mean square of the successive differences; **nuLF PSD**: normalized power spectrum of the 0.04 to 0.15Hz band; **nuHF PSD**: normalized power spectrum of the 0.15 to 0.4 Hz band; **nuLF/nuHF**: ratio between the bands; **SD1 axis**: non-linear feature associated with short-term changes; **SD2 axis:** non-linear feature associated with long-term changes and **SD1/SD2**: ratio between axis.
