# Supplementary material for: Autonomic nervous system response to remote ischemic conditioning: heart rate variability assessment
Source: BMC Cardiovasc Disord. 2019 Sep 9;19:211. doi: 10.1186/s12872-019-1181-5 (PMC6734354; doi:10.1186/s12872-019-1181-5)
Supplement: Supplementary file 6 — Table S4. Young population analysis for the first and last 10 min and occlusion and non-occlusion intervals. For the first and last 10 min analysis, the mean values are presented as well as a comparison between them and the p-value for the Wilcoxon signed-rank test. For the occlusion and non-occlusion interval analysis, the mean values are presented as well as a comparison between them and the p-value for the Wilcoxon signed-rank test. (PDF 60 kb) [file 12872_2019_1181_MOESM6_ESM.pdf]

Supplementary Table 4 - Young population analysis for the first and last 10 minutes and occlusion and non-occlusion intervals

**Young**					
		First 10 minutes Mean	Last 10 minutes Mean	First vs Last	Wilcoxon signed-rank test p-value: Before - After pairs
**Time Features**	Mean R-R Interval (ms)	808,285	819,949	Last higher	0,285
	Median R-R Interval (ms)	811,325	822,175	Last higher	0,359
	pNN50 (%)	16,039	16,830	Last higher	0,508
	rMSSD (ms)	38,061	39,792	Last higher	0,445
**Frequency Features**	nuLF PSD (%)	46,850	48,150	Last higher	0,444
	nuHF PSD (%)	29,700	27,450	First higher	0,102
	nuLF/nuHF ratio	1,791	1,985	Last higher	0,241
**Non-linear Features**	SD1 axis (ms)	26,912	28,136	Last higher	0,445
	SD2 axis (ms)	86,350	92,111	Last higher	0,508
	SD1/SD2	0,308	0,307	First higher	0,878

Mean R-R Interval: mean value of the time difference between beats; **Median R-R Interval**: median value of the time difference between beats; **pNN50**: percentage of beats that differ more than 50ms from the previous; **rMSSD**: root mean square of the successive differences; **nuLF PSD**: normalized power spectrum of the 0.04 to 0.15Hz band; **nuHF PSD**: normalized power spectrum of the 0.15 to 0.4 Hz band; **nuLF/nuHF**: ratio between the bands; **SD1 axis**: non-linear feature associated with short-term changes; **SD2 axis:** non-linear feature associated with long-term changes and **SD1/SD2**: ratio between axis.

Supplementary Table 4 (cont.)

		Non-occlusion intervals Mean	Occlusion intervals Mean	Non-Occlusion vs Occlusion	Wilcoxon signed-rank test p-value: Non-Occlusion - Occlusion
**Time Features**	Mean R-R Interval (ms)	816,104	816,448	Occlusion higher	0,959
	Median R-R Interval (ms)	818,261	819,087	Occlusion higher	0,878
	pNN50 (%)	16,602	16,042	Non-occlusion higher	0,241
	rMSSD (ms)	39,240	38,422	Non-occlusion higher	0,093
**Frequency Features**	nuLF PSD (%)	47,632	48,800	Occlusion higher	0,575
	nuHF PSD (%)	28,142	28,800	Occlusion higher	0,878
	nuLF/nuHF ratio	1,924	2,066	Occlusion higher	0,799
**Non-linear Features**	SD1 axis (ms)	27,746	27,168	Non-occlusion higher	0,093
	SD2 axis (ms)	89,882	87,798	Non-occlusion higher	0,333
	SD1/SD2	0,310	0,308	Non-occlusion higher	0,721

Mean R-R Interval: mean value of the time difference between beats; **Median R-R Interval**: median value of the time difference between beats; **pNN50**: percentage of beats that differ more than 50ms from the previous; **rMSSD**: root mean square of the successive differences; **nuLF PSD**: normalized power spectrum of the 0.04 to 0.15Hz band; **nuHF PSD**: normalized power spectrum of the 0.15 to 0.4 Hz band; **nuLF/nuHF**: ratio between the bands; **SD1 axis**: non-linear feature associated with short-term changes; **SD2 axis:** non-linear feature associated with long-term changes and **SD1/SD2**: ratio between axis.
